# Factors Affecting the Use of Private Outpatient Services among the Adult Population in Malaysia

**DOI:** 10.3390/ijerph192013663

**Published:** 2022-10-21

**Authors:** Jailani Anis-Syakira, Suhana Jawahir, Nurul Salwana Abu Bakar, Sarah Nurain Mohd Noh, Nurul Iman Jamalul-Lail, Normaizira Hamidi, Sondi Sararaks

**Affiliations:** 1 Centre for Health Outcomes Research, Institute for Health Systems Research, Ministry of Health Malaysia, Shah Alam 40170, Malaysia; 2 National Institutes of Health, Ministry of Health Malaysia, Shah Alam 40170, Malaysia; 3 Centre for Health Equity Research, Institute for Health Systems Research, Ministry of Health Malaysia, Shah Alam 40170, Malaysia; 4 Centre for Health Policy Research, Institute for Health Systems Research, Ministry of Health Malaysia, Shah Alam 40170, Malaysia; 5 Centre for Health Services Research, Institute for Health Systems Research, Ministry of Health Malaysia, Shah Alam 40170, Malaysia; 6 Centre for Health Quality Research, Institute for Health Systems Research, Ministry of Health Malaysia, Shah Alam 40170, Malaysia; 7 Director Office, Institute for Health Systems Research, Ministry of Health Malaysia, Shah Alam 40170, Malaysia

**Keywords:** health care, utilisation, outpatient, private, predictors

## Abstract

The proportion of Malaysians of all ages who use private outpatient services has dropped over time, highlighting the overstretched condition of public outpatient facilities compared to their private counterparts. This paper aims to determine the prevalence of outpatient care, characteristics of outpatient care users by sector, and the factors affecting the utilisation of private outpatient services among the adult population of Malaysia using Andersen’s behavioural model. Data from the National Health Morbidity Survey 2019 (NHMS 2019), a nationwide survey, were analysed. Logistic regression analysis was performed to explore the association of predisposing (locality, age, sex, ethnicity, education level, and marital status), enabling (working status, health care coverage, and household income), and need factors (perceived and evaluated needs) with the use of private outpatient services. Variables with a statistical significance ≤ 0.25 in the univariate regression analysis were included in the final multivariable logistic regression analysis. A total of 11,674 respondents, estimated to represent 22.4 million adults aged 18 years and above in Malaysia, were included for analysis. Overall, 8.3% of the adult population of Malaysia used outpatient care and 33.9% used the private sector. Those living in urban areas (OR = 1.80, 95% CI = 1.02, 3.18), non-Malays (OR = 1.74, 95% CI = 1.04, 2.93), those working (OR = 2.47, 95% CI = 1.48, 4.10), those with employer coverage (OR = 4.73, 95% CI = 2.79, 8.01), and those with health problems (OR = 2.26, 95% CI = 1.26, 4.05) were more likely to utilise private outpatient services. Those who self-rated their health status as fair (OR = 0.54, 95% CI = 0.33, 0.91) and who had diabetes, hypertension, or hypercholesterolemia (OR = 0.56, 95% CI = 0.31, 1.02) were less likely to utilise private outpatient services. The predisposing and enabling factors were associated with the use of private outpatient services, and the need factors were strong predictors of private outpatient care utilisation among adults. Understanding the factors associated with the utilisation of private outpatient services could aid in the development of effective initiatives designed to enhance outpatient care access among the population of Malaysia and balance the burden of outpatient care provision on the public and private sector.

## 1. Introduction

Malaysia’s health care system has undergone significant restructuring since the country’s independence in 1957 [1]. Since the Alma Ata Declaration in 1978, the first reform began with the public primary health care services and has accelerated since [2]. Primary care services are the foundation of the healthcare system and highly essential for the delivery of health care, as they serve as the initial point of care for the general public for the treatment of basic illnesses, routine check-ups, and vaccination services, as well as first-aid for injuries [3]. They also serve as the basis for referrals to secondary and tertiary health care services, with secondary care referring to intermediate health care consultation or services provided by specialists to referred patients, and tertiary care referring to advanced treatment, complex surgeries, and inpatient care [3]. In Malaysia, outpatient care constitutes both the primary and secondary care that do not require an overnight hospital stay [4].

Malaysia, an upper-middle-income country with a population of about 32 million people, has a unique two-tier health care system consisting of the public and private sectors. Public health providers offer a full range of health services, including outpatient curative, preventative, promotive, and rehabilitative services, whereas private health providers primarily provide curative care and diagnostic health services in the outpatient setting [3]. The public health services are funded through general taxation, while the private sector provides services on a fee-for-service basis [3]. The Ministry of Health (MOH), the public sector’s main provider of outpatient services [5], has strived to make outpatient care affordable and accessible to all. Outpatient care in the public sector is heavily subsidised and accessible to all individuals for a nominal fee or for free. Malaysian citizens pay MYR 1 (USD 0.24) for a general outpatient consultation and MYR 5 (USD 1.22) for a specialist consultation, while non-citizens pay MYR 15 (USD 3.64) and MYR 60 (USD 14.54) [3], respectively. Furthermore, the fees are waived for specific groups, such as government employees, pensioners, senior citizens, and physically, mentally, and economically disadvantaged individuals [3], as well as for specific services, such as child immunisation and antenatal and postnatal care [6].

According to the NHMS 2019 findings, the Malaysian population make 2.7 outpatient visits to a doctor per year, as measured by the number of outpatient consultations per person per year [4]. Malaysia has similar levels of outpatient utilisation to neighbouring countries, such as Vietnam (2.3 visits), Thailand (2.1 visits), and Singapore (1.7 visits) [7], but lower utilisation compared to the average of Economic Cooperation and Development (OECD) countries (6.9 visits), as well as other middle-income countries such as Mongolia (6.1 visits) and Sri Lanka (5.1 visits) [7]. 

As of December 2019, there were 144 public hospitals and special medical institutions, as well as 3210 public clinics, scattered throughout the country. The private sector, on the other hand, has 208 licensed private hospitals and 7988 registered medical clinics, which are concentrated in urban areas [8]. According to the trend in outpatient service utilisation, the proportion of Malaysians of all ages who use private outpatient services has dropped over time, declining from 54.2% in 2011 [9] and 39.9% in 2015 [10] to 35.7% in 2019 [4], which has resulted in a greater burden on the already overstretched public outpatient facilities.

Various theoretical models have been developed to explain health care utilisation [11,12,13,14,15]. In our study, Andersen’s behavioural model was adopted to identify factors affecting private outpatient service utilisation. It is the model most widely used to analyse the use of health services, which posits that the utilisation of health services is affected by predisposing and enabling factors of its use and the need for care [16].

Many studies worldwide have reported the prevalence of outpatient care and predictors of private outpatient service utilisation [17]. However, to the best of our knowledge, to date, no published study has reported the factors affecting private outpatient care utilisation in Malaysia using data from a nationwide household survey. Our study intends to fill the knowledge gap in this area so as to aid in planning and policy formulation in order to improve outpatient care access among the Malaysia population and shift the burden of outpatient care provision from public to private outpatient healthcare facilities. Thus, this study aimed to determine the prevalence of outpatient care, characteristics of the outpatient care users by the sector of health care facilities, and the factors affecting the private outpatient service utilisation among the adult population of Malaysia using Andersen’s behavioural model.

## 2. Materials and Methods

We analysed data of adults aged 18 years and above from the National Health and Morbidity Survey (NHMS) 2019, a nationwide, cross-sectional population survey of morbidity, health status, and health-seeking behaviour among the non-institutionalised general population of Malaysia. A random two-stage, stratified, proportional-to-size sampling design was employed to select a nationally representative sample, with the sampling frame provided by the Department of Statistics of Malaysia. All 13 states and 3 federal territories were included in the study, covering both urban and rural areas. The stratification was carried out by state (primary stratum) and by urban or rural area formed within the primary stratum (secondary stratum). A total of 11,674 adults aged 18 years and over were eligible and invited to participate in this survey. The data collection was conducted from July to October 2019 via face-to-face interview using a bilingual (Malay/English), structured, pre-tested and pilot-tested questionnaire [18,19]. The overall response rate for this survey was 83.4%. A detailed methodology and sampling design of the survey is available elsewhere [4].

### 2.1. Dependent Variables

Outpatient care utilisation was measured by the question “In the last 2 weeks, have you received any outpatient care?”, with the “yes” or “no” response choices. Those who answered “do not know” or “refuse to answer” were categorised as “no”. Those who replied “yes” were then asked, “Is that place owned by the government or private?”, with the choices of “government” or “private” as responses. The place referred to the outpatient health facilities used by the respondents. Of those who utilised more than one facility, only the first reported health facility was analysed, as the majority of respondents (96.1%) visited only one health facility. Government facilities will be referred to as public facilities in this study. Of the 11,674 adult respondents, about 1207 reported that they have utilised outpatient services and were included for further analysis.

### 2.2. Determinants of Private Outpatient Care Utilisation

#### 2.2.1. Predisposing Factors

The predisposing factors studied were locality, age, sex, ethnicity, education level, and marital status. Locality was categorised as “urban” or “rural” based on the population density of the area [20]. Age, initially measured as a continuous variable in years, was coded into three groups: “18–34”, “35–59”, and “60 and above”. Self-reported ethnicity indicated self-reported identification with one of the following groups: Malay, Chinese, Indian, Aborigines, Bumiputera of Sabah, Bumiputera of Sarawak, and Others. “Non-Malay” (i.e., Chinese, Indian, Aboriginal, Bumiputera of Sabah, Bumiputera of Sarawak, and Others) were collapsed to improve the distribution. Education was categorised as “no formal education”, “primary”, “secondary”, and “tertiary”. Marital status was classified into “single” and “married”. Single, widowed, and divorced were collapsed into “single” in the analysis. 

#### 2.2.2. Enabling Factors

The enabling factors included working status, health coverage, and household income. For working status, respondents were classified as “active” (employed) and “inactive” (unemployed, retiree, student, and housewife). Health coverage was defined as having financial coverage for health care and categorised into two mutually exclusive categorical variables: “covered by government” (possession of either a government guarantee letter (GL), pensioner card, or government-specific health fund) and “covered by employer” (covered by either employer-provided medical benefits, employer-sponsored health insurance, or a panel clinic). Household income was calculated based on the total monthly household income, and respondents were classified according to quintiles, with the first (Q1) and last quintile (Q5) representing the poorest and richest individuals of the population, respectively. 

#### 2.2.3. Need Factors

Perceived and evaluated needs were included as proxy measures for the need factors. Among the perceived needs, reported health problems and self-rated health status were included. Reported health problems were measured by the question, “In the last two weeks, did you experience any of the following health problems, such as fever, sore throat, difficulty in swallowing, running nose or blocked nose, cough, and other symptoms of acute health problems?”, with “yes” or “no” as response choices. Self-rated health status was assessed using a five-point scale (excellent, good, fair, poor, very poor) and the question, “How would you rate your health status?” In the analysis, the responses were grouped into three categories: “excellent–good”, “fair”, and “very poor–poor”. The evaluated need was assessed based on the presence of diabetes, hypertension, or hypercholesterolemia, as diagnosed by a doctor or health care professional. Respondents were asked the following questions: “Have you ever been told by a doctor or Assistant Medical Officer that you have diabetes?”; “Have you ever been told by a doctor or Assistant Medical Officer that you have high blood pressure?”; and “Have you ever been told by a doctor or Assistant Medical Officer that you have high cholesterol?”, to which the answer was either a “yes” or “no”.

### 2.3. Statistical Analysis

Descriptive statistics were used to illustrate the characteristics of the respondents and users of private and public outpatient services. Univariate and multivariable regression analyses were employed to investigate the associations between the predisposing, enabling, and need factors with the utilisation of private outpatient services. Variables obtaining a statistical significance ≤ 0.25 in the univariate regression analysis were included in the final multivariable regression analysis. Reference groups were chosen based on common standards with the exception of groups with homogeneous characteristics. 

An appropriate survey design was applied in all the analyses to account for the sampling method and study design, with statistical significance of *p*-values of less than 0.05, using STATA version 13 (Stata Corp, College Station, TX, USA). Crude odds ratios (OR) and adjusted ORs with 95% confidence intervals (CI) were reported. The goodness of fit was assessed using receiver operating characteristic curves and areas under the curve (AUC). The AUC values lie between 0 and 1, according to which an AUC of 0.9–1.0 is considered excellent, 0.8–0.9 very good, 0.7–0.8 good, 0.6–0.7 sufficient, 0.5–0.6 bad, and less than 0.5 is not useful [21]. The multicollinearity was examined using VIF (variance inflation factor). A VIF value greater than 10 indicates the presence of a multicollinearity problem [22].

## 3. Results

A total of 11,674 respondents, estimated to represent 22.4 million adults of 18 years and over in Malaysia, were included in the analysis. Females constituted 50.1%, urban dwellers 75.6%, those aged 18 to 34 years old 44.4%, Malays 51.3%, married individuals 62.9%, those with secondary education 46.8%, those working 62.1%, and those who self-rated their health as good to excellent 78.5% (Table 1).

Overall, 8.3% of the adult population of Malaysia used outpatient care. Of this group, 33.9% used private outpatient care, while 66.1% used public outpatient care. Table 2 and Table 3 describe the characteristics of the respondents who utilised outpatient care in general, as well as private and public facilities for outpatient care. Those who utilised private outpatient care had substantially different characteristics, except for sex, ethnicity, marital status, and possession of health coverage by government. The majority of private sector users in Malaysia were residing in urban areas (39.3%), aged between 18 to 34 years old (41.3%), those with tertiary education (54.9%), working individuals (48.1%), individuals covered by employer health care coverage (69.3%), among the 20% of richest individuals in the population (56.5%), those with health problems (38.8%), those who rated their health status as good to excellent (42.0%), and those who had no diabetes, hypertension, or hypercholesterolemia (42.2%).

After controlling for all other variables, factors that showed significant results in determining the utilisation of outpatient care in private facilities were locality, ethnicity, working status, and possession of health coverage by an employer, as well as the need factors of reported health problems, self-rated health status, and the presence of diabetes, hypertension, or hypercholesterolemia (Table 4). The likelihood of using private outpatient care was higher among those residing in urban areas (OR = 1.80, 95% CI = 1.02, 3.18), among non-Malays (OR = 1.74, 95% CI = 1.04, 2.93), those working (OR = 2.47, 95% CI = 1.48, 4.10), with employer coverage (OR = 4.73, 95% CI = 2.79, 8.01), and with health problems in the two weeks prior to the survey (OR = 2.26, 95% CI = 1.26, 4.05). Those who self-rated their health status as fair (OR = 0.54, 95% CI = 0.33, 0.91) and who had either diabetes, hypertension, or hypercholesterolemia (OR = 0.56, 95% CI = 0.31, 1.02) had lower odds of private outpatient care utilisation compared with those who rated their health status as good to excellent and those without diabetes, hypertension, or hypercholesterolemia, respectively. 

The AUC for this model was 0.7735, indicating a good fit. The multicollinearity analysis showed that all variables had VIFs of less than 5, ranging from 1.28 to 3.75. This indicates that multicollinearity was unlikely.

## 4. Discussion

Globally, many operations and procedures that historically required inpatient care and services are being phased out in favour of outpatient care and may now be safely conducted in an outpatient setting [23]. Previous surveys have shown an overall decline in inpatient service utilisation in Malaysia by 1.7% over the last eight years [4,9,10]. Regardless, outpatient service utilisation among the Malaysian population of all ages has also shown a declining trend from 12.6% in 2011 to 9.0% percent in 2019 [4,9,10] despite the increases in the population size, ageing population, and non-communicable diseases (NCDs). The well-publicised issue of long waiting times and congestion in public health facilities may influence the decision of the population to utilise outpatient services less often, which might be a plausible reason for the declining overall outpatient service utilisation in Malaysia [24]. 

Based on Andersen’s behavioural model [15], health service utilisation is highly dependent on predisposing, enabling, and need factors. In our study, we found that living in an urban area is significantly associated with private outpatient care utilisation, which is similar to the findings from other studies [25,26,27]. The accessibility factor drives private utilisation among the urbanites, since most of the private outpatient facilities are concentrated in urban areas [28,29]. In Malaysia, there is an unequal distribution of private outpatient clinics, since there are no regulations governing where the facilities can operate [30]. Thus, to ensure an even distribution of areas where outpatient facilities may operate, and to improve access to the outpatient services, a legislative framework regulating the locations, opening, licensing, and operation of private clinics needs to be formulated and implemented. This framework could discourage the overcrowding in private clinics in one particular area and promote the opening of facilities in rural parts of the country by controlling where the facilities can operate [31]. This study showed that non-Malays exhibited greater odds of visiting private outpatient care facilities than the Malays. After Malays, the Chinese constitute the second largest ethnic group in Malaysia, accounting for 22.8% of the population [32], and the majority of them live in urban areas [33]. Considering that private health services are more readily available in urban areas, this could explain why the Chinese showed a higher likelihood of using the private healthcare sector than Malays. Conversely, more Malays use public outpatient care, which is in line with the fact that the bulk of Malaysia’s government employees are Malays [34,35], and that government pensioners and employees, as well as their dependents, are exempted from paying fees for health services provided by the public sector [36].

Our study reported that the enabling factors of working status and possession of employer coverage had significant associations with the private outpatient care utilisation among adults in Malaysia. Those who were working were twice as likely to use private outpatient care compared with those who were not economically active. Similarly, other published literature also reported that those who are unemployed are more likely to use public primary health care [37]. Working adults with private coverage usually have higher odds of utilising private health clinics, as private companies in Malaysia offer health coverage for their employees in the form of a panel doctor or health insurance for private care, which also covers the utilisation of private outpatient care [38]. This finding is similar to those of other studies conducted elsewhere, according to which those with private health insurance are more likely to choose private care [39,40]. A pre-payment mechanism may be one of the strategies used to provide people with the option to choose between public and private services. Policymakers could provide incentives, such as tax relief, to employers who provide employee health benefits so as to encourage employees to use more private facilities and alleviate the load on the public sector, in addition to providing the option to choose between public and private services in order to seek care.

Among the Malaysia population, need factors seem to be the push and pull factors for utilising outpatient facilities. Those with health problems in the two weeks prior to the survey were found to be twice as likely to utilise private outpatient care compared with those without recent health problems, and those who self-rated their health status as “fair” showed lower odds of private outpatient care utilisation compared with those who rated their health status as “good” to “excellent”. The Ministry of Health has an ‘open door’ policy regarding outpatient services [3]; however, the population can choose between public and private services. Those who have recently experienced health problems and rate their health as “good” to “excellent” may be more aware of their health and the negative consequences of delaying treatment, thus choosing private services over public outpatient care due to convenience and shorter waiting times [3].

Those who had either diabetes, hypertension, or hypercholesterolemia showed lower odds of private outpatient care utilisation compared with those without diabetes, hypertension, or hypercholesterolemia. This might be attributed to the primary care initiative in Malaysia, which covers regular follow-up visits for patients with long-term health concerns for the purpose of continuous monitoring [41]. Despite the fact that Malaysia practices a dual healthcare delivery system, the public health system, which is extensively subsidised by the government, is responsible for the majority of chronic disease management and treatment [42]. As a result, the bulk of follow-up cases occur in public facilities, which may explain the lower odds of private outpatient care utilisation among those with diabetes, hypertension, or hypercholesterolemia in Malaysia. 

This finding is consistent with a study in Albania, where those who self-rated their health as poor and suffered from chronic conditions were more likely to utilise the public facilities [43]. However, contrary to our findings, a study from Korea reported that those with less comorbidities and who attend primary care clinics for managing conditions other than simple or minor diseases were more likely to use public facilities [44].

The population’s reliance on public services is apparent and continues to grow over time. Findings from previous surveys showed an increasing trend of utilisation of outpatient services provided by public facilities among the Malaysia population, increasing from 48.8% in 2011 and 60.1% in 2015 to 64.3% in 2019. This, as a result, will add pressure on the already overburdened public sector [45]. Strategic public–private partnerships offer a way forward to ensure that health care is more accessible and affordable by leveraging the vast resources of private general practitioner networks and private hospitals. Additionally, the current government’s financing scheme, which provides financially disadvantaged citizens with access to private facilities [46,47], may be further improved and expanded. As the existing structure requires patients to pay for their own treatment if they continue treatment at a private facility, the benefit package could allow patients to continue receiving the required treatment at the same private facility without needing to pay out of their own pocket.

The idea of providing social insurance in Malaysia has been discussed for many years by the policymakers and the stakeholders. The Peka B40 and MySalam schemes were finally introduced in the Malaysian healthcare system in the hope of providing much-needed relief to lower income groups. In Georgia, Medical Insurance for the Poor (MIP) was rolled out nationwide, and several studies reported that the program improved the financial protection of the targeted individuals and expedited the access to outpatient and inpatient care for acute patients. The MIP program had a positive equity impact by offering better financial protection to the poorest [48]. In the United Kingdom, the Stay Well Pharmacy campaign was launched in 2018 to promote the use of community pharmacists for the care of minor ailments in order to lessen outpatient visits [49]. Should Malaysia implement similar public health efforts to support the management of minor ailments by private primary service providers, such as community pharmacies and private general practitioners, this could ease congestion at public health clinics for the benefit of sicker patients.

Previous research found an association between the variables of gender and income and the utilisation of health services [17]. Another study conducted by Awoke et al. found that there was a significant association of private healthcare utilisation with income but not gender [50]. This study, however, found no association between private outpatient care utilisation and gender or income. 

## 5. Conclusions

Overall, 8.3% of the adult population of Malaysia used outpatient care. Of this group, two-thirds used public outpatient care. Predisposing and enabling factors were associated with the use of private outpatient services, and need factors were strong predictors of private outpatient care utilisation among adults in Malaysia. Understanding the factors associated with the utilisation of private outpatient services could aid in the development of effective initiatives so as to enhance outpatient care access among the population of Malaysia and balance the burden of outpatient care provision on the public and private sectors.

## Figures and Tables

**Table 1:** Characteristics of the respondents (N = 11,674).

Characteristics	Count, *n* (Unweighted)	Estimated Population, *n* (Weighted)	Percentage (%)
Overall	11,674	22,366,558	100.0
***Predisposing factors***			
**Locality**			
Urban	7015	16,911,101	75.6
Rural	4659	5,455,457	24.4
**Age (years)**			
18–34	3729	9,931,466	44.4
35–59	5441	9,134,887	40.8
60 and above	2504	3,300,204	14.8
**Sex**			
Male	5517	11,168,723	49.9
Female	6157	11,197,835	50.1
**Ethnicity**			
Malay	7642	11,484,897	51.3
Non-Malay	4032	10,881,661	48.7
**Education level**			
No formal education	679	1,218,140	5.4
Primary	2540	4,402,673	19.7
Secondary	5554	10,893,547	48.7
Tertiary	2862	5,762,962	25.8
Unknown	39	89,236	0.4
**Marital status**			
Single	3738	8,283,226	37.0
Married	7927	14,060,869	62.9
Unknown	9	22,463	0.1
***Enabling factors***			
**Working status**			
No	4884	8,462,011	37.8
Yes	6781	13,887,591	62.1
Unknown	7	11,967	0.1
**Covered by government**			
No	8617	18,048,920	80.7
Yes	2914	4,024,734	18.0
Unknown	143	292,904	1.3
**Covered by employer**			
No	9363	17,173,327	76.8
Yes	2168	4,900,327	21.9
Unknown	143	292,904	1.3
**Household income**			
Poorest	1594	2,908,033	13.0
Q2	2086	3,973,610	17.8
Q3	2292	4,327,135	19.3
Q4	2890	5,892,335	26.3
Richest	2725	5,108,377	22.8
Unknown	87	157,067	0.7
***Need factors***			
**Reported health problems**			
Yes	2747	4,660,921	79.0
No	8900	17,668,308	20.8
Unknown	27	7329	0.2
**Self-rated health status**			
Good—Excellent	8751	17,556,981	78.5
Fair	2558	4,167,054	18.6
Very Poor—Poor	284	457,119	2.0
Unknown	81	185,404	0.8
**Presence of DM, HPT, or HCHOL**			
Yes	3311	4,905,633	76.0
No	8142	17,001,975	21.9
Unknown	221	458,950	2.1

Q: quintile, DM: diabetes, HPT: hypertension, HCHOL: hypercholesterolemia. Malay included Orang Asli.

**Table 2:** Prevalence of the outpatient care users (N = 11,674).

Characteristics	Count, *n* (Unweighted)	Estimated Population, *n* (Weighted)	Percentage, % (95% CI) (Weighted)
Overall	1207	1,859,823	8.3 (7.5–9.2)
***Predisposing factors***			
**Locality**			
Urban	695	1,353,438	8.0 (7.1–9.1)
Rural	512	506,385	9.3 (8.0–10.8)
**Age (years)**			
18–34	275	625,119	6.3 (5.3–7.5)
35–59	542	756,992	8.3 (7.3–9.4)
60 and above	390	477,711	14.5 (12.3–16.7)
**Sex**			
Male	478	770,470	6.9 (6.0–8.0)
Female	729	1,089,352	9.7 (8.6–11.0)
**Ethnicity**			
Malay	817	1,033,587	9.0 (8.1–10.0)
Non-Malay	390	826,236	7.6 (6.4–9.0)
**Education level**			
No formal education	108	148,054	12.2 (8.9–16.5)
Primary	332	483,764	11.0 (9.3–12.9)
Secondary	486	755,550	6.9 (6.1–7.9)
Tertiary	276	459,009	8.0 (6.5–9.8)
Unknown	5	13,445	15.1 (4.0–42.8)
**Marital status**			
Single	347	566,448	6.8 (5.8–8.1)
Married	860	1,293,375	9.2 (8.3–10.2)
***Enabling factors***			
**Working status**			
No	606	855,619	10.1 (8.9–11.5)
Yes	600	1,003,210	7.2 (6.3–8.3)
Unknown	1	994	8.3 (10.1–44.5)
**Covered by government**			
No	801	1,359,585	7.5 (6.7–8.4)
Yes	399	491,867	12.2 (10.4–14.4)
Unknown	7	8371	2.9 (1.2–6.9)
**Covered by employer**			
No	980	1,425,878	8.3 (7.5–9.2)
Yes	220	425,573	8.7 (7.1–10.6)
Unknown	7	8371	2.9 (1.2–6.9)
**Household income**			
Poorest	211	326,543	11.2 (9.2–13.7)
Q2	217	323,282	8.1 (6.6–10.0)
Q3	248	340,252	7.9 (6.5–9.4)
Q4	250	413,242	7.0 (5.8–8.5)
Richest	272	445,810	8.7 (7.1–10.7)
Unknown	9	10,694	6.8 (2.6–16.4)
***Need factors***			
**Reported health problems**			
Yes	904	1,420,211	30.5 (27.7–33.4)
No	301	434,159	2.5 (2.1–2.9)
Unknown	2	5453	14.6 (3.6–43.8)
**Self-rated health status**			
Good—Excellent	654	1,042,524	5.9 (5.2–6.7)
Fair	449	678,075	16.3 (14.2–18.6)
Very Poor—Poor	97	126,946	27.8 (21.0–35.8)
Unknown	7	12,278	6.6 (2.2–18.5)
**Presence of DM, HPT, or HCHOL**			
No	561	987,690	5.8 (5.1–6.6)
Yes	633	852,035	17.4 (15.3–19.6)
Unknown	13	20,097	4.4 (1.8–10.1)

Q: quintile, CI: confidence interval, DM: diabetes, HPT: hypertension, HCHOL: hypercholesterolemia. Malay included Orang Asli.

**Table 3:** Characteristics of the outpatient care users by sector of health care facilities (N = 1207).

Characteristics	Proportion of Outpatient Service Utilisation						
	Used Private Facilities			Used Public Facilities			*p*-Value
	Count, *n* (Nonweighted)	Estimated Population, *n* (Weighted)	Percentage, % (95% CI) (Weighted)	Count, *n* (Unweighted)	Estimated Population, *n* (Weighted)	Percentage, % (95% CI) (Weighted)	
Overall	328	630,567	33.9 (29.1–39.0)	879	1,229,255	66.1 (61.0–70.9)	-
***Predisposing factors***							
**Locality**							
Urban	225	531,384	39.3 (33.2–45.7)	470	822,054	60.7 (54.3–66.8)	<0.001
Rural	103	99,183	19.6 (14.1–26.6)	409	407,202	80.4 (73.5–85.9)	
**Age (years)**							
18–34	100	258,209	41.3 (33.0–50.2)	175	366,910	58.7 (49.8–67.1)	<0.001
35–59	167	288,176	38.1 (31.4–45.2)	375	468,816	61.9 (54.8–68.6)	
60 and above	61	84,183	17.6 (12.8–23.8)	329	393,529	82.4 (76.2–87.2)	
**Sex**							
Male	139	288,546	37.5 (30.0–45.6)	339	481,925	62.6 (54.4–70.0)	0.1522
Female	189	342,022	31.4 (26.5–36.8)	540	747,331	68.6 (63.2–73.5)	
**Ethnicity**							
Malay	205	313,165	30.3 (25.3–35.8)	612	720,422	69.7 (64.2–74.7)	0.1188
Non-Malay	123	317,402	38.4 (29.9–47.7)	267	508,834	61.6 (52.3–70.1)	
**Education level**							
No formal education	17	34,432	23.3 (12.4–39.3)	91	113,623	76.7 (60.7–87.6)	<0.0001
Primary	67	109,777	22.7 (16.7–30.1)	265	373,987	77.3 (69.9–83.3)	
Secondary	126	234,332	31.0 (24.7–38.1)	360	521,219	69.0 (61.9–75.3)	
Tertiary	117	252,015	54.9 (45.4–64.1)	159	206,995	45.1 (35.9–54.6)	
Unknown	1	12	0.001 (0.0001–0.01)	4	13,432	99.9 (98.9–99.9)	
**Marital status**							
Single	100	188,062	33.2 (26.4–40.8)	247	378,386	66.8 (59.2–73.7)	0.8181
Married	228	442,506	34.2 (28.6–40.3)	632	850,869	65.8 (59.7–71.4)	
***Enabling factors***							
**Working status**							
No	96	147,933	17.3 (13.2–22.3)	510	707,687	82.7 (77.7–86.8)	<0.0001
Yes	232	482,635	48.1(41.1–55.2)	368	520,575	51.9 (44.8–58.9)	
Unknown	0	-	-	1	994	100.0	
**Covered by government**							
No	230	463,096	34.1 (28.2–40.4)	571	896,488	65.9 (59.6–71.8)	0.4245
Yes	98	167,471	34.1 (27.2–41.6)	301	324,396	66.0 (58.4–72.8)	
Unknown	0	-	-	7	8371	100.0	
**Covered by employer**							
No	184	335,821	23.6 (19.2–28.6)	796	1,090,057	76.5 (71.5–80.8)	<0.0001
Yes	144	294,746	69.3 (59.7–77.4)	76	130,827	30.7 (22.6–40.3)	
Unknown	0	-	-	7	8371	100.0	
**Household income**							
Poorest	33	70,087	21.5 (14.5–30.7)	178	256,456	78.5 (69.4–85.5)	<0.0001
Q2	37	81,290	25.2 (17.1–35.3)	180	233,608	74.9 (64.7–82.9)	
Q3	66	104,161	30.6 (22.5–40.1)	182	246,478	69.4 (59.9–77.5)	
Q4	65	117,447	28.4 (20.3–39.3)	185	298,377	71.6 (61.7–79.7)	
Richest	124	252,083	56.5 (45.5–67.0)	148	193,727	43.5 (33.0–54.4)	
Unknown	3	5499	51.4 (13.2–88.1)	6	5195	48.6 (11.9–86.8)	
***Need factors***							
**Reported health problems**							
Yes	284	550,565	38.8 (33.6–44.2)	620	869,645	61.2 (55.8–66.4)	0.0002
No	44	80,002	18.4 (11.5–28.1)	257	354,157	81.6 (71.9–88.5)	
Unknown	0	-	-	2	5453	100.0	
**Self-rated health status**							
Good—Excellent	218	437,374	42.0 (35.1–49.1)	436	605,150	58.1 (50.9–64.9)	<0.0001
Fair	91	156,946	23.2 (18.0–29.2)	358	521,129	76.9 (70.8–82.0)	
Very Poor—Poor	18	35,082	27.6 (15.8–43.7)	79	91,863	72.4 (56.3–84.2)	
Unknown	1	1165	9.5 (1.0–5.1)	6	11,112	90.5 (48.9–99.0)	
**Presence of DM, HPT, or HCHOL**							
No	200	416,578	42.2 (35.9–48.7)	361	571,112	57.8 (51.3–64.1)	0.0001
Yes	121	202,355	23.8 (17.8–30.9)	512	649,680	76.3 (69.1–82.2)	
Unknown	7	11,634	57.9 (20.5–88.0)	6	8463	42.1 (12.0–79.5)	

Q: quintile, CI: confidence interval, DM: diabetes, HPT: hypertension, HCHOL: hypercholesterolemia. Malay included Orang Asli.

**Table 4:** Crude and adjusted ORs with 95% CIs for the utilisation of private outpatient services among those who utilised outpatient care.

Variables	Univariate Logistic Regression		Multiple Logistic Regression	
	Crude Odds Ratio, OR (95% CI)	*p* > |t|	Adjusted Odds Ratio, OR (95% CI)	*p* > |t|
***Predisposing factors***				
**Locality**				
Urban	**2.65 (1.65, 4.26)**	<0.0001	**1.80 (1.02, 3.18)**	0.042
Rural (ref)	1.00		1.00	
**Age (years)**				
18–34 (ref)	1.00		1.00	
35–59	0.87 (0.57, 1.33)	0.528	1.39 (0.82, 2.35)	0.219
60 and above	**0.30 (0.18, 0.51)**	<0.0001	1.52 (0.69, 3.38)	0.298
**Sex**				
Male (ref)	1.00		1.00	
Female	0.76 (0.53, 1.11)	0.153	1.21 (0.81, 1.80)	0.354
**Ethnicity**				
Malay (ref)	1.00		1.00	
Non-Malay	1.43 (0.91, 2.26)	0.119	**1.74 (1.04, 2.93)**	0.036
**Education level**				
No formal education	0.67 (0.30, 1.53)	0.345	0.91 (0.39, 2.15)	0.835
Primary education	0.65 (0.42, 1.02)	0.063	0.90 (0.51, 1.58)	0.709
Secondary education (ref)	1.00		1.00	
Tertiary education	**2.70 (1.68, 4.35)**	<0.0001	0.98 (0.54, 1.77)	0.945
**Marital status**				
Not Married (ref)	1.00			
Married	1.05 (0.71, 1.54)	0.818	-	-
***Enabling factors***				
**Working status**				
No (ref)	1.00		1.00	
Yes	**4.44 (2.92, 6.74)**	<0.0001	**2.47 (1.48, 4.10)**	0.001
**Covered by government**				
No (ref)	1.00			
Yes	1.00 (0.66, 1.51)	0.998	-	-
**Covered by employer**				
No (ref)	1.00		1.00	
Yes	**7.31 (4.6, 11.6)**	<0.001	**4.73 (2.79, 8.01)**	<0.001
**Household income**				
Poorest (ref)	1.00		1.00	
Q2	1.22 (0.66, 2.30)	0.516	0.74 (0.35, 1.54)	0.415
Q3	1.6 (0.85, 3.05)	0.140	0.75 (0.36, 1.54)	0.434
Q4	1.45 (0.75, 2.80)	0.264	0.50 (0.23, 1.06)	0.069
Richest	**4.76 (2.53, 8.98)**	<0.0001	1.17 (0.51, 2.70)	0.709
***Need factors***				
**Reported health problems**				
Yes	**2.80 (1.63, 4.8)**	<0.0001	**2.26 (1.26, 4.05)**	0.007
No (ref)	1.00		1.00	
**Self-rated health status**				
Good—Excellent (ref)	1.00		1.00	
Fair	**0.42 (0.27, 0.64)**	<0.0001	**0.54 (0.33, 0.91)**	0.020
Very poor—Poor	0.53 (0.25, 1.11)	0.092	1.39 (0.63, 3.06)	0.409
**Presence of DM, HPT, or HCHOL**				
Yes	**0.43 (0.28, 0.65)**	<0.0001	0.56 (0.31, 1.02)	0.059
No (ref)	1.00		1.00	

Q: quintile, CI: confidence interval, OR: odds ratio, ref: reference category, DM: diabetes, HPT: hypertension, HCHOL: hypercholesterolemia. Malay included Orang Asli. Statistically significant *p*-values (<0.05) are shown in bold. Multicollinearity was unlikely (VIF < 10). Variables obtaining a statistical significance ≤ 0.25 in the univariate regression analysis were included in the final multivariable regression analysis. The overall fit of the model for binary logit was checked using the weighted area under receiver operating characteristic (ROC) curve, 0.7735. The fit of the models was considered based on the area under the curve.

## Data Availability

To protect the privacy of the respondents, the data set that supports the findings of this article is not publicly available. Requests for data can be addressed to the Head of the Centre for Biostatistics & Data Repository, National Institutes of Health, Ministry of Health Malaysia, on reasonable request and with the permission of the General Director of Health, Malaysia.
